# 

**Published:** 1863-03

**Authors:** 


### CONTENTS.

ORIGINAL ESSAYS AND COMMUNICATIONS.

Elements of Professional success. By B. P. AYDELOTT, M. D., D D.... 101
Dental Fees and Etiquette. By A. BENEDICT.... 114
Does Breathing Through the Mouth Injure the Teeth? By GEO. F. FOOTE, M. D.... 121
Theory and Practice. By W. H. ATKINSON.... 125
Address. By J. MANSFIELD.... 129

PROCEEDINGS OF SOCIETIES.

Nineteeth Annual Meeting of the Mississippi Valley Association of Dental Surgeons.... 131

CORRESPONDENCE.

A Lusus Naturæ. By E. Collins.... 134

SELECTIONS.

Dentition and its Derangements. By A. JACOBI, M. D.... 135
Case of Hæmorrhage from a branch of the Anterior Palatine Artery. By H. T. Kempton. 141
Hygienic and Therapeutic Relations of Good Teeth.... 142

EDITORIAL.

The Patent Rubber.... 144
Personal.... 146
Dental Materials.... 146
Leslie's Reguline Gold.... 146

